# Comparative Analysis of Whole-Genome and Methylome Profiles of a Smooth and a Rough *Mycobacterium abscessus* Clinical Strain

**DOI:** 10.1534/g3.119.400737

**Published:** 2019-11-12

**Authors:** Chiranjibi Chhotaray, Shuai Wang, Yaoju Tan, Amjad Ali, Muhammad Shehroz, Cuiting Fang, Yang Liu, Zhili Lu, Xingshan Cai, H. M. Adnan Hameed, Md Mahmudul Islam, Goverdhan Surineni, Shouyong Tan, Jianxiong Liu, Tianyu Zhang

**Affiliations:** * State Key Laboratory of Respiratory Disease, Guangzhou Regenerative Medicine and Health Guangdong Laboratory, Guangzhou Institutes of Biomedicine and Health, Chinese Academy of Sciences, Guangzhou 510530, China,; † University of Chinese Academy of Sciences, Beijing 100049, China,; ‡ State Key Laboratory of Respiratory Disease, Department of Clinical Laboratory, Guangzhou Chest Hospital, Guangzhou 510095, China,; § Atta-ur-Rahman School of Applied Biosciences (ASAB), National University of Sciences and Technology (NUST), H-12, Islamabad 44000, Pakistan, and; ** Institute of Physical Science and Information Technology, Anhui University, Hefei 230027, China

**Keywords:** *Mycobacterium abscessus*, SMRT sequencing, comparative genomics, whole genome sequencing, methylation, single nucleotide variation

## Abstract

*Mycobacterium abscessus* is a fast growing *Mycobacterium* species mainly causing skin and respiratory infections in human. *M. abscessus* is resistant to numerous drugs, which is a major challenge for the treatment. In this study, we have sequenced the genomes of two clinical *M. abscessus* strains having rough and smooth morphology, using the single molecule real-time and Illumina HiSeq sequencing technology. In addition, we reported the first comparative methylome profiles of a rough and a smooth *M. abscessus* clinical strains. The number of N4-methylcytosine (4mC) and N6-methyladenine (6mA) modified bases obtained from smooth phenotype were two-fold and 1.6 fold respectively higher than that of rough phenotype. We have also identified 4 distinct novel motifs in two clinical strains and genes encoding antibiotic-modifying/targeting enzymes and genes associated with intracellular survivability having different methylation patterns. To our knowledge, this is the first report about genome-wide methylation profiles of *M. abscessus* strains and identification of a natural linear plasmid (15 kb) in this critical pathogen harboring methylated bases. The pan-genome analysis of 25 *M. abscessus* strains including two clinical strains revealed an open pan genome comprises of 7596 gene clusters. Likewise, structural variation analysis revealed that the genome of rough phenotype strain contains more insertions and deletions than the smooth phenotype and that of the reference strain. A total of 391 single nucleotide variations responsible for the non-synonymous mutations were detected in clinical strains compared to the reference genome. The comparative genomic analysis elucidates the genome plasticity in this emerging pathogen. Furthermore, the detection of genome-wide methylation profiles of *M. abscessus* clinical strains may provide insight into the significant role of DNA methylation in pathogenicity and drug resistance in this opportunistic pathogen.

*M. abscessus* is a major non-tuberculous *Mycobacterium* (NTM) species causing pulmonary infections in human. The treatment of this emerging pathogen is a major challenge because it is resistant to most of the effective drugs along with disinfectants (Medjahed *et al.* 2010; Leung and Olivier 2013) whereas only few antibiotics show bacteriostatic effect (Maurer *et al.* 2104). Based on phylogenetic analysis of housekeeping genes (*rpoB*, erythromycin ribosome methyltransferase gene, and macrolide resistance-related gene), *M. abscessus* is categorized into three subspecies, *M. abscessus* subsp *abscessus*, *M. abscessus* subsp *massiliense*, and *M. abscessus* subsp *bolletii* (Adékambi *et al.* 2006; Bastian *et al.* 2011; Macheras *et al.* 2011). Importantly, *M. abscessus* subsp. *abscessus* is a “nightmare” bacterium, more resistant and prevalent than other subspecies (Nessar *et al.* 2012). Additionally, the clinical features and treatment outcomes are different for patients infected with *M. abscessus* complex (Koh *et al.* 2011; Harada *et al.* 2012). It is very important to understand the drug resistance mechanism and genetic relatedness of *M. abscessus* clinical isolates at genetic level. The traditional molecular methods like multilocus sequencing typing (MLST), pulsed-field gel electrophoresis (PFGE), and variable number tandem repeat (VNTR) are used to determine the genetic relatedness of *M. abscessus* isolates. However, the uses of these methods are limited to typing of strains within the sub species of *M. abscessus*. To overcome this limitation, whole-genome sequencing approach can be facilitated for understanding the pathogenicity of this emerging pathogen at the genome level. Ripoll *et al.* (2009) have sequenced the whole genome of *M. abscessus* ATCC 19977^T^ strain in 2009. Later on, several research groups have reported the whole-genome sequences of *M. abscessus* clinical strains (Pang *et al.* 2013; Sekizuka *et al.* 2014; Caverly *et al.* 2016). The second generation sequencing has become a popular technology to sequence the microbial genomes for understating the population structure and identify the single nucleotide variations (SNVs) as well as large-scale deletions in the genome of bacterial pathogens. Similarly, the third generation sequencing technology is also used for sequencing of whole genome and detecting the methylation modification in the genomes (Flusberg *et al.* 2010). Single molecule real-time sequencing (SMRT) is one of the third generation sequencing technologies that enable to identify N6-methyladenine (6mA), N4-methylcytosine (4mC) and 5-methylcytosine (5mC) modifications in bacterial genomes which facilitates in exploring the epigenetic modification in bacteria. The methylome profile is very important in any bacterial species because DNA methylation is involved in various physiological processes of bacteria. Moreover, DNA methylation process is a part of restriction-modification (R-M) systems which has an important role in bacterial defense mechanism (Vasu and Nagaraja 2013). Notably, methylation protects *M. tuberculosis* from hypoxia and stress condition (Shell *et al.* 2013). Recently, SMRT sequencing technology is well adapted in the field of mycobacterial research (Chhotaray *et al.* 2018) and the technique was used to study the methylome of some mycobacterial species (Zhu *et al.* 2016; Phelan *et al.* 2018). Zhu *et al.* (2016) have reported the methylation of 12 *Mycobacterium tuberculosis* complex (MTBC) by using SMRT sequencing technology and three DNA methyltransferases (MTases) responsible for m6A modification were identified. Additionally, Phelan *et al.* (2018) have reported the diversity of methylation in *M. tuberculosis* and *M. africanum* by using SMRT sequencing and identified lineage-specific methylated motifs and strain-specific mutations. However, methylome analysis of any NTM has not been reported yet. We sought to assess the utility of SMRT sequencing technology to study the methylome of *M. abscessus* clinical isolates.

In the current study, we report the complete genome sequences of two *M. abscessus* clinical strains (having smooth and rough morphology) isolated from the patients who had a pulmonary infection. The comparative genomic studies of the two clinical strains were performed, which shows significant variations among them at genome level. Additionally, this is the first study showing the complete methylome analysis of NTM by using SMRT sequencing technology. We have identified 6mA and 4mC modification in the smooth and rough *M. abscessus* clinical strains and analyzed the variation in methylation sites in their genomes. Moreover, this is the first report that a 15 kb linear natural plasmid exists in *M. abscessus* clinical strain and its methylation is reported here.

## Materials and Methods

### Drug susceptibility testing

The *M. abscessus* clinical strain GZ002 (Mab^S^) with smooth morphology (Guo *et al.* 2016) and another clinical stain GZ0 1 (Mab^R^) showing rough morphology were collected from Guangzhou Chest Hospital, China. Drug susceptibility test was performed by the broth microdilution method in 96-well plates as described previously by clinical and laboratory standards institute (CLSI) (Woods *et al.* 2011). The eleven anti-TB drugs tested, isoniazid, rifampin, streptomycin, ethambutol, levofoxacin, clarithromycin, amikacin, linezolid, clofazimine, and ethionamide were listed in the Supplemental Material, Table S4. The MIC was determined as the lowest concentration of a drug that prevents visible bacterial growth.

### Bacterial DNA isolation and whole-genome sequencing

The isolates were grown in Middlebrook 7H9 liquid medium containing 10% oleic acid-albumin-dextrose-catalase (OADC) and 5% glycerol for 5 days and then streaked on 7H10 agar medium. The single colonies were inoculated into 7H9 liquid medium. The genomic DNA was extracted from both clinical isolates by using standard protocols (van Soolingen *et al.* 2001). Genomic DNA was fragmented, and then 20 kb DNA fragments were taken for preparation of SMRTbell DNA template libraries. DNA fragments were end repaired and ligated with universal hairpin adapters and the subsequent steps were followed as per the manufacture’s instruction to prepare SMRTbell library. The obtained library was sequenced in PacBio RSII SMRT instrument (McCarthy 2010) and HGAP (version 4.0) pipeline was used to assemble PacBio’s reads (Chin *et al.* 2013). The validation of the quality of the assembly and final genome sequence was determined by using the Quiver consensus algorithm (Chin *et al.* 2016). Finally, the genome was circularized by trimming the ends of assembled sequences. The genomes of two strains were re-sequenced by using Illumina HiSeq to resolve the errors found during SMRT sequencing. The paired-end libraries were prepared from the genomic DNA and were sequenced by Illumina HiSeq instrument (Illumina, San Diego, CA, USA). The details of the library preparation and bioinformatics analysis are mentioned in the supplementary material (Supplementary sheet 1). The Illumina raw reads were trimmed at the percentage of bases with Phred value greater than 20 (less than 1% probability of error). The alignment software BWA (version 0.7.12) was used to align the clean data generated from Mab^S^ and Mab^R^ to the *M. abscessus* ATCC 19977^T^ reference strain genome sequence (NC_010397.1) (Li and Durbin 2009). The alignment result was corrected by using Picard (https://broadinstitute.github.io/picard/) and GATK (DePristo *et al.* 2011). The statistics of raw data generated from Illumina and SMRT sequencing are mentioned in the Table S5-8 and Table S9-10 respectivly.

The non-coding RNAs like rRNA and tRNA were predicted by RNAmmer (version 1.2) (Lagesen *et al.* 2007), tRNAscan-SE (version 1.3.1) (Lowe and Chan 2016) respectively whereas mapping Rfam (version 12.2) (Nawrocki *et al.* 2015) method was applied to predict other non-coding RNAs. Prodigal (version 3.02, prokaryote) is used for the prediction of protein-coding genes in Mab^S^ and Mab^R^ genomes (Hyatt *et al.* 2010). The coding genes were annotated with the National Center for Biotechnology Information (NCBI) nr database by Diamond (Buchfink *et al.* 2014). The functional annotation of genes was performed by GO (Gene Ontology) database (Harris *et al.* 2004), and the KEGG (Kyoto Encyclopedia of Genes and Genomes) database was used for pathways annotation (Kanehisa and Goto 2000). The genes encoding proteins were classified on functional categories through a COG (Clusters of Orthologous Groups) database (Tatusov *et al.* 2003). The circos (version 0.69) software was used to display genome sequence in circular plot. In this study, we have identified a linear plasmid from Mab^S^ which was confirmed by gelelectrophoresis. The sequence of the identified plasmid was BLASTed in NCBI to check the percentage of identity with other strain of *M. abscessus*. Clustered regularly interspaced short palindromic repeats (CRISPRs) elements were searched using the CRISPRfinder web tool (Grissa *et al.* 2007).

### Comparative genomics

For the identification of insertion or deletion in the respective genomes, the sequences of Mab^R^ (CP034191), Mab^S^ (CP034181), and *M. abscessus* reference genome (NC_010397.1) were aligned using BLAST Ring Image Generator (BRIG v0.95 and NCBI BLAST+) with upper and lower threshold value 90% and 70%, respectively (Alikhan *et al.* 2011). To visualize the insertions and deletions in Mab^R^ (CP034191) and Mab^S^ (CP034181), the genomes were aligned with the reference genome of *M. abscessus* ATCC 19977^T^ strain (NC_010397.1). Similarly, Mab^R^ (CP034191) was used as a reference to align and visualize the insertions and deletions in Mab^S^ with respect to Mab^R^.

The pan-genome analysis was performed by using the predicted proteome of 25 *M. abscessus* strains. In order to explore the pan-genome of *M. abscessus*, the bacterial pan-genome analysis (BPGA) pipeline (version 1.3) was used (Chaudhari *et al.* 2016). BPGA performed pre-processing step to prepare sequence data and then clustering was done by using USEARCH (Edgar 2010) with a default threshold value of 50% sequence identity. The clustered output was analyzed to obtain gene presence/absence binary matrix file (pan-matrix) which was subsequently used for pan-genome profile calculations with a total of 20 iterations as well as pan-genome based phylogeny. To perform core genome-based phylogenetic analysis, core proteins of all *M. abscessus* genomes were extracted. Concatenated amino acid sequences of the core proteome were aligned using MUSCLE (Edgar 2004), and a phylogenetic tree was constructed using the Neighbor Joining method (Saitou and Nei 1987). The phylogenetic trees were annotated using the interactive tree of life (iTOL) version 3 (Letunic and Bork 2016).

To detect the SNV at the genome level, the reads of each strain were mapped against the reference strain genome (NC_010397.1) using Samtools (version 1.1) (Li *et al.* 2009) and the UnifiedGenotyper module from GATK (DePristo *et al.* 2011). Before SNV analysis, mapping results were processed to remove duplication by picard (V1.119) (https://broadinstitute.github.io/picard/). SNVs were filleted based on the parameters like the minimum SNV quality score 10 and minimum read depth 10×. The detected mutations were annotated by Annovar software (Wang *et al.* 2010). The reliability of the detected SNV was evaluated by summarizing the number of reads of each SNV site in every sample along with the distance of the adjacent SNV sites. Based on the information of annotated gene provided in the database, the software correlated the mutation information with the gene information to achieve the interpretation of the mutation site.

### Genome wide base modification and motif analysis

The detection of genome wide base modification was performed by SMRTlink. Base modification analysis was performed based on normalization of the kinetics values. Inter pulse duration (IPD); the primary metric was used for base modification analysis. The quality value (QV) score threshold was set at 50 for genome-wide methylation pattern analysis. Pacific Bioscience’s SMRTPortal was used for identification of the position of modified bases (Feng *et al.* 2013) and sequences of methylated motifs were identified as previously described (Furuta *et al.* 2014). The data obtained from SMRT sequencing analysis containing each motif’s methylation site, methylation score, type of methylation, and all locations of a discovered motif (File Xls S2).

### Plasmid extraction From Mab^S^ and Mab^R^ strain

Briefly, 15 mL of Mab^S^ and Mab^R^ strains were grown to 1.0 at OD_600_. The bacterial culture was then centrifuged at 3800× g for 10 min and pellet was resuspended in 1 mL of TE buffer. 100 μL of lysozyme (10 mg/mL) and 100 μL proteinase K (200 mg/mL) was added and incubated in a shaker at 37°, 180 rpm, for 24 hr. The mixture was vigorously vertexed and pellet down. The plasmid was extracted using Maogen Kit (HiPure Plasmid Maxi Kit).

### Data availability

All sequence data were deposited in the NCBI database. Accession numbers of genome sequences are CP034181 for *M. abscessus* GZ002 (Mab^S^) and CP034191 for *M. abscessus* GZ0 1 (Mab^R^) and accession number of the pMabS_GZ002 plasmid is CP034180. The raw data of Mab^S^ and Mab^R^ were submitted in NCBI having SRA accession number PRJNA504433 and PRJNA495001 respectively. Supplementary sheet 1 contains details of Illumina sequencing library preparations and bioinformatics analysis of sequencing data. Figure S1 contains the KEGG classification annotation statistics of Mab^S^ and Mab^R^ whereas Figure S2 provides the COG classification annotation statistics of Mab^S^ and Mab^R^. Figure S3 and Figure S4 display the pan-genome and core genome phylogeny of 25 complete genome sequences of *M. abscessus* species respectively. Figure S5 provides the gelelectrophoresis of plasmids extracted from *M. abscessus* clinical strains. Figure S6 contains the maps representing the circular visualization of insertions and deletions in Mab^R^ (CP034191) and Mab^S^ (CP034181). Table S1 provides the annotations and related data for linear plasmid pMabS_GZ002 gene. Table S2 contains the information about the variation in methylation sites in the genes of interest between two clinical strains of *M. abscessus*. Table S3.1, Table S3.2, and Table S3.2, give the information about the gene content in the inserted and deleted regions Reference *vs.* Mab^R^, Reference *vs.* Mab^S^, Mab^S^ *vs.* Mab^R^, respectively. Table S4 contains the information about the drug susceptibility of *M. abscessus* isolates. Table S5 contains statistics of PF data generated by Illumina sequencing. Table S6 provides the statistics of clean data after quality control. Table S7 contains the proportion of statistics of the clean data compared to PF data after quality control. Table S8 gaves the genome alignment statistics of two *M. abscessus* clinical strains obtained from Illumina sequencing. Table S9 gives the raw reads statistics of two of *M. abscessus* clinical strains generated from SMRT sequencing. Table S10 provides the genome assembly statistics of two *M. abscessus* clinical strains obtained from SMRT sequencing. File Xls S1 contains result of structural variation analysis. File Xls S2 gives the information about the identified SNVs, each motif’s methylation site, methylation score, type of methylation, and all locations of discovered motifs in both strains, motif information of pMabS_GZ002 plasmid, identified methyltransferase in both strains. File Xls S3 contains the statistics of the 25 available complete genomes of *M. abscessus* used for pan-genome analysis. File Xls S4 privids the statistics number of accessory genes, unique genes as well as exclusively absent genes determined among the *M. abscessus* species. MabS.h5 and MabR.h5 contain the raw files of methylome analysis of Mab^S^ and Mab^R^, respectively. Supplemental material available at figshare: https://doi.org/10.25387/g3.10251044.

## Results

### Drug susceptibility testing

The two *M. abscessus* clinical isolates were tested for antimicrobial susceptibility using the broth microdilution method as described previously (Woods *et al.* 2011). The result of antibiotic susceptibility test showed that resistance level of rough strain to clofazimine was a little more than that of the smooth strain but we didn’t observe colony morphology affect the susceptibilities of *M. abscessus* to other anti-TB drugs (Table S4).

### Genome assembly and annotation

**Figure 1:**
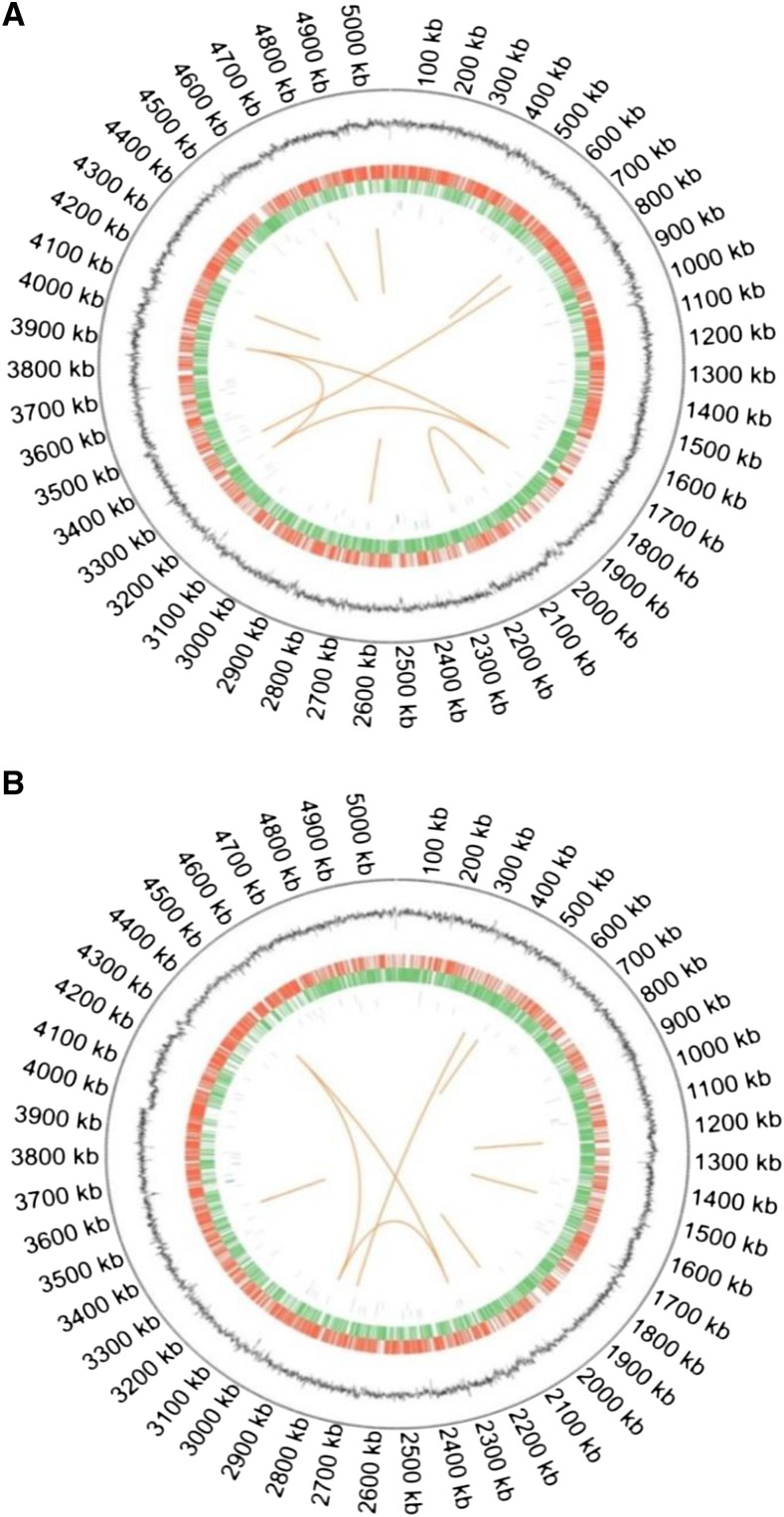
Circular chromosome maps for Mab^R^ (A) and Mab^S^ (B), generated by using circos (version 0.69) software. The circular plot has five levels. From outside to inside, the first is size of chromosome indicated in kb, the (second) inner black histogram represents the is G+C content, the third is positive strand genes (marked in red), the fourth is negative strand genes (marked in green), the fifth is positive strand ncRNA (marked in blue), the sixth is negative strand ncRNA (marked in purple) and the seventh shows long repeats (>100bp).

The genomes of two *M. abscessus* clinical strains were first sequenced by SMRT sequencing technology (Flusberg *et al.* 2010) then re-sequenced by using Illumina HiSeq sequencing. The high quality reads having Phred quality score of 20 were obtained from sequencing data of both clinical strains. After the multiple filtering of raw data, a total of 31184792 and 14125608 numbers of reads obtained from Mab^S^ and Mab^R^, respectively were mapped to the reference genome (Table 1). The average length of sequence reads was 148.72 bp for both strains. The genomes were circularized; the size of Mab^S^ genome is 5067231 bp in length, with an average G+C content of 64.41% (Figure 1, Table 1) whereas the genome size of Mab^R^ is 5075529 bp with an average G+C content of 64.70% (Figure 1). There are 4963 and 5001 protein-coding genes were identified in Mab^S^ and Mab^R^, respectively. Moreover, 9 rRNAs and 46 tRNAs were predicted for both assembled genomes (Table 1). In addition, 37 other non-coding RNAs were identified form Mab^R^ and Mab^S^ (Table 1), respectively. The genes of both strains were categorized into six groups in the KEGG database (Figure S1). The distribution of functional COG categories was grouped into 21 categories for both strains (Figure S2).

**Table 1:** Genome assembly and annotation statistics of the two of M. abscessus clinical strains

Description	Mab^S^	Mab^R^
Genome Size (bp)	5067231	5075529
Coverage (%)	100	100
Mean depth	86.23	149.40
G+C Content (%)	64.41	64.70
Number of coding genes	4963	5001
Total bases for all genes (bp)	4712088	4705875
Minimum length of genes (bp)	99	99
Maximum length of genes (bp)	24327	23520
Average length of genes (bp)	949.44	940.99
Total Number of non coding RNAs	92	92
Number of rRNAs	9	9
Number of tRNAs	46	46
Number of other ncRNAs.	37	37

**Table 2:** Types of single-nucleotide variations (SNVs) identified in M. abscessus isolates compared with reference strain

Strain	Total No. of SNVs	Non-Synonymous	Synonymous	Stop codon gain	Stop codon lost
Mab^R^	1358	385	752	2	2
Mab^S^	8	6	1	0	1

Interestingly, a 15203 bp linear plasmid was identified in Mab^S^ having G+C content of 67.55% and 15 protein coding genes were found in the plasmid (Table S1). For further confirmation, the plasmid was extracted from the Mab^S^ strain and the gel electrophoresis result showed a band at around 15 kb (Figure S5). We designated this plasmid as pMabS_GZ002. Notably, plasmid carries a mercury resistance operon and putative resolvase/invertase/recombinase similar to *M. abscessus* ATCC 19977^T^ plasmid pMAB23. However, further analysis may be needed to support the plasmid linearity. We didn’t find any CRISPR elements in the two genomes.

### Comparative genomics study

The two annotated genomes were compared with the *M. abscessus* reference genome (NC_010397.1) to study the structural variations among the respective genomes. The result of this analysis revealed that a 60 bp insertion was found in the genome of Mab^S^ compared with *M. abscessus* reference genome (File Xls S1, Table S3.1-3.3, and Figure S6). Interestingly, 13 insertions and 8 deletions were identified in the genome of Mab^R^ with respect to the reference genome. We compared the genomes of both clinical isolates which showed 7 insertions and 10 deletions in the genome of Mab^R^ (File Xls S1, Table S3.1-3.3, and Figure S6). This indicates that Mab^S^ and *M. abscessus* reference genome (NC_010397.1) are similar to each other.

**Figure 2:**
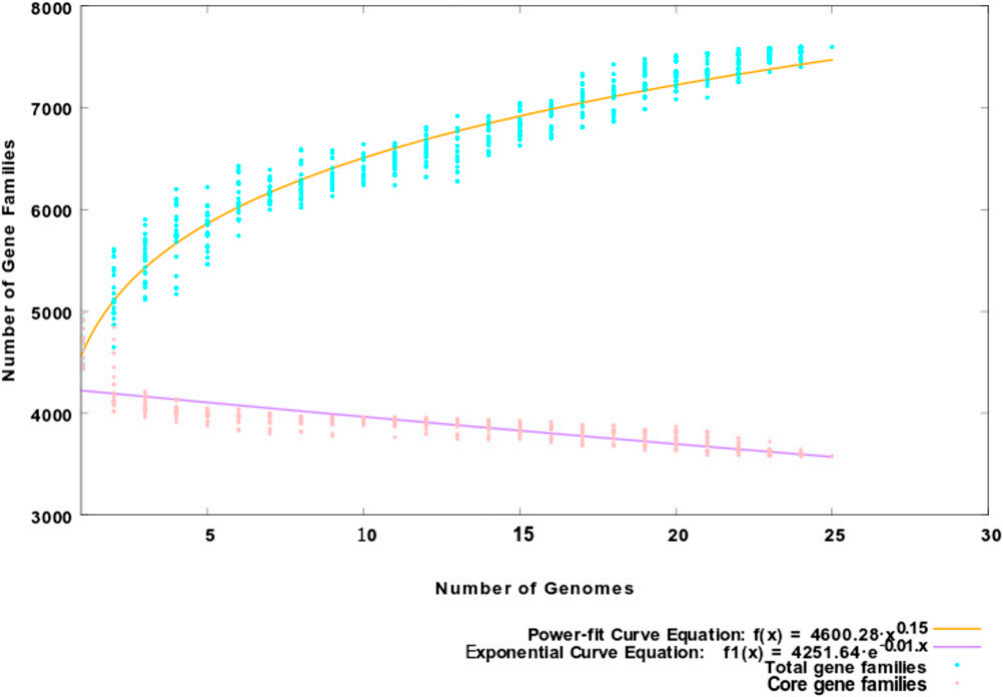
Prediction of *M. abscessus* pan- and core-genome. The exponential and power-fit models of core and pan genes are shown in the plot by purple and green lines, respectively. The relevant equations used for computing core and pan-genome are also included for visualization. The analysis (plots) indicated that the *M. abscessus* (25) species having an open pan genome which comprises of 7596 gene clusters.

Pan-genome analysis was performed by using the predicted protein sequences of the 25 strains *M. abscessus*. The core genes were defined as homologous genes that are present in all strains. The specific or unique genes have obtained that present in only one of the strains that are refered as “strain-specific” whereas the sum of all genes is called pan genes (pan-genome). The statistics of the 25 available complete genomes such as G+C contents, genome sizes, strain names, Biosample number, Bioproject number, etc. are provided in File Xls S3. The pan-genome of *M. abscessus* species was found to comprise of 7596 protein clusters and the number of core protein clusters was found to be 3585. The core/pan ratio was calculated as 0.47195 which projected that core form ∼47.195% of the pan-genome size. The expansion parameter ’b’ was calculated to be 0.150554, *i.e.*, somewhat greater than zero, which indicates that the pan-genome of this species retains the ability to accommodate more unique genes into its gene pool and thus increase the size of its pan-genome. Therefore, the pan-genome although is now open but may be closed soon because availability of more complete genomes of this species in the future so the nature of its pan-genome (open or close) can be elucidated with greater confidence to get a realistic picture. This trend is also visible in the pan-core plot (Figure 2). The number of accessory genes, unique genes as well as exclusively absent genes were also determined (File Xls S4). Mab^R^ strain contributed total 20 unique genes toward the pan-genome while the Mab^S^ strain contributed total 16 unique genes. Also, Mab^R^ seems to lack fifty-eight genes which are found in other 24 genomes; meanwhile, a total of two genes was exclusively absent in Mab^S^ strain. Pan-phylogeny revealed that Mab^R^ strain is evolutionarily related to strain FLAC046 from USA (Figure S3). Further, the clade of these two strains is linked to another clade containing strain NOV0213 (Russia), FLAC029 (USA) and FLAC028 (USA). However, the core-phylogeny projected that Mab^R^ is evolutionary closest to G220 previously isolated from China, followed by NOV0213 isolated from Russia (Figure S4). On the other hand, Mab^S^ is evolutionary closest to strain 7C isolated previously from Malaysia, followed by FLAC013 strain from the USA. These evolutionary relationships of Mab^S^ with related strains are corroborated by the core genome phylogeny. The results of this analysis showed that a high level of genomic diversity was evident in this species, as expected due to the open nature of the pan-genome.

We have identified 1358 and 8 SNVs in Mab^R^ and Mab^S^ respectively, based on mapping of sequence reads of each strain to the reference strain. There are 385 nsSNVs and 752 synonymous SNVs (sSNVs) were detected in Mab^R^ strain (Table 3). Importantly, two genes were observed as truncated (*MAB_2069* and *MAB_2074*) and the other two genes have lost the stop codon (*MAB_0280* and *MAB_2073*) due to SNV in Mab^R^. Out of 8 SNVs, 6 nsSNVs and only one (*MAB_0280*) sSNV found in Mab^S^ strain where as SNV causes the lost of a stop codon in one gene but no gene found to be truncated due to SNV (File Xls S2).

**Table 3:** Single-nucleotide variations (SNVs) cause the change in amino acid that is common in both sequenced strains in comparison to reference strain ATCC 19977^T^

Position	SNV	Gene	Amino acid changes	Protein
280242	T-C	*MAB_0280*	Stop-Q	Hypothetical protein
1150461	A-T	*MAB_1137c*	E-V	Putative membrane protein, MmpL family
2106418	C-T	*MAB_2106c*	T-I	Probable conserved lipoprotein LppL
2583761	C-T	*MAB_2537c*	H-Y	Putative pyruvate ecarboxylase

In order to investigate the nsSNVs detected in the genes of Mab^R^, we specially focused on the majority of genes harboring the nsSNVs (File Xls S2). There are 62, 41, 22 and 20 nsSNVs were present within the *MAB_2100*, *MAB_2073*, *MAB_2099*, and *MAB_2074* respectively. The highest number of nsSNV was found in *MAB_2100* encoding putative plasmid replication initiator protein. In addition, nsSNVs were found within genes encoding putative monooxygenase, hypothetical cell division FtsK/SpoIIIE protein, bacteriophage protein, hypothetical protein, recombinase, and transcriptional regulator. We have identified 4 genes (*MAB_0280*, *MAB_1137c*, *MAB_2106c*, and *MAB_2537c*) containing nsSNVs which are common to both clinical strains (Table 3).

### Genome wide methylome analysis of two M. abscessus clinical strains

SMRT sequencing technology enables to detect methylation modification in the genome. This facilitated us to analyze and determine the positions of modified DNA bases in two respective clinical strains of *M*. *abscessus* having smooth and rough colony morphology, respectively. The 5mC modifications are not detected because DNA was not treated with Tet1 oxidation prior to sequencing. Both *M. abscessus* strains possessed more 4mC type of base modification than m6A modification. Totals of 6381 (88.6%) and 817 (11.3%) modified bases having a QV score of 50 were detected as 4mC and 6mA respectively, in Mab^S^.

**Figure 3:**
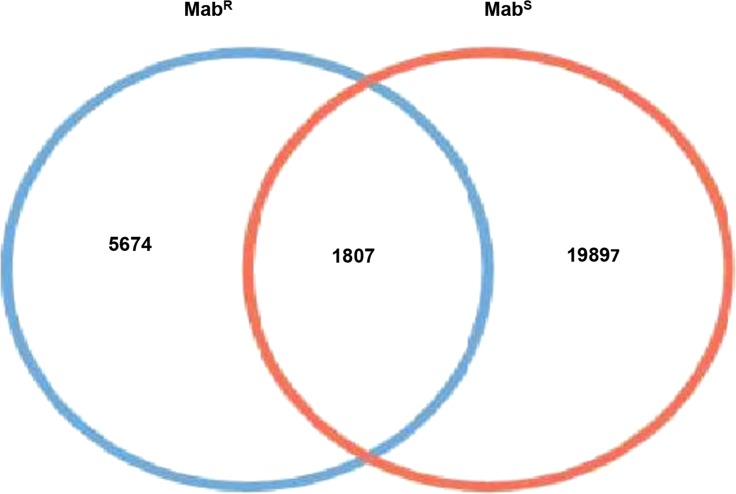
The Venn diagram represents the comparison of DNA modified bases in Mab^S^ and Mab^R^ strain which shows the Mab^S^ strain is having more modified bases (19897) than Mab^R^ strain (5674) whereas 1807 detected DNA modified bases are common to both strains.

One putative motif “VSGGCCKVNB” was identified for m4C type of modification in Mab^S^ but we didn’t get any motif for 6mA type modification. Additionally, two motifs (“VVGGCCB” and “GTNNBVNB”) were detected for “modified_base” which indicates that methylation was expected but below the significance threshold during the initial kinetics analysis (Table 4). In the case of Mab^R^, we found 2960 (85%) 4mC and 496 (14%) 6mA methylated bases with a QV score 50. Interestingly, the total number of methylated bases detected in Mab^S^ was considerably two-fold higher than that of Mab^R^. In contrast to Mab^S^, only one motif “VVGGCCKS” was identified for “modified_base” but we didn’t find any specific motifs for 6mA and 4mC type of modification in Mab^R^ (Table 4). Additionally, we explored the genome wide variation in methylome between both *M. abscessus* clinical strains. There were 1807 common modified base sites detected in the genomes of both strains. Furthermore, 19897 and 5674 modified base sites were identified that are specific to Mab^S^ and Mab^R^, respectively (Figure 3). We have detected 34 4mC methylated bases in the plasmid (pMabS_GZ002) obtained from Mab^S^ (File Xls S2). However, we didn’t find any 6mA methylated bases in the plasmid. Furthermore, there are 95 and 96 MTases identified in smooth and rough strain, respectively by genome analysis (File Xls S2).

**Table 4:** Methylation motifs detected for M. abscessus clinical strains

*M*. *abscessus* clinical strain	Detected Modification sequence	Modification type	No. of occurrences of motifs in the genome	No. of instances of motifs detected as modified	% Motifs methylated	Mean IPD Ratio
Mab^S^	VVGGCCB	modified_base	45741	4549	9.94	3.0841002
	VSGGCCKVNB	m4C	16457	870	5.28	3.0538025
	GTNNBVNB	modified_base	321226	3166	0.98	2.330288
Mab^R^	VVGGCCKS	modified_base	21145	1805	8.5	3.9614415

### Association of methylome with M. abscessus antibiotic resistance, intracellular survival and Glyco-Peptido-Lipids (GPLs) locus

Methylation modifications were identified in the genes encoding antibiotic target-modifying enzymes and intracellular survival factors in both *M. abscessus* clinical strains. Our study identified 4 of 5 genes encoding antibiotic-modifying enzymes having methylation modification. Interestingly, it was found that position of methylation site (m4C) in *MAB_2297* [erm (41)] gene encoding erythromycin ribosome methylase is different in both the clinical strains where as one m4C methylation site was detected in each *MAB_2385* (3′’-O-phosphotransferase) and *MAB_2875* (β-lactamase) gene of Mab^S^ but there is no such type of methylation found in Mab^R^ (Table S2). In addition, *MAB_1496c* encoding flavin monooxygenase containing one m4C modification site in Mab^R^ in contrast to Mab^S^.

The actual molecular mechanisms of intracellular survival of *M. abscessus* remain unclear. However, some genes were identified that are associated with intra-amoebal and/or intra-macrophage survival by transposon library screening (Laencina *et al.* 2018). Here, we have identified the genes associated with intracellular survival have methylation modifications (m4C and m6A). The study of Laencina *et al.* (2018) has shown that *esx-4* genes of *M. abscessus* containing transposon insertion which leads to reduced intra-amoebal replication. Our methylome study showed that the position of the methylation sites in *esx-4* genes varies in both clinical strains. Importantly, transposon insertion in *MAB_3757* gene of *esx*-4 locus showed a 75% reduction in intra-amoebal replication has 3 methylation sites identified in Mab^S^ in contrast to Mab^R^ site (Table S2). Similarly, *MAB_0628* gene encoding EspI - secretion protein is showing a severe defect in intra-amoebal survival has 8 methylation sites in Mab^S^ where as one common methylation site found in both strains (Table S2). Furthermore, we have identified methylation sites in two genes of GPLs locus in both clinical strains; these genes were down regulated in rough variant compared to smooth variant strain in the previous study (Pawlik *et al.* 2013). The *MAB_4099c* gene of this locus which encodes non-ribosomal peptide synthetase has 25 and 4 methylation sites in Mab^S^ and Mab^R^ respectively whereas only one methylation site found common to both strains. Similarly, 9 and 6 methylation sites were detected in the *MAB_4098c* gene of GPLs locus in Mab^S^ and Mab^R^ (Table S2).

## Discussion

Whole genome sequencing approach followed by the comparative genomic analysis has provided useful insights into the genome dynamics of *M. abscessus* species. In this study, the genetic variations in *M. abscessus* clinical strains were explored. We believe this is the first study to analyze the respective methylomes of *M. abscessus* clinical strains. The genetic information of *M. abscessus* is necessary for understanding its complex lifestyle in natural conditions. The genome of this pathogen is containing a large number of genes encoding antibiotic-target-modifying enzymes responsible for intrinsic resistance to a wide range of antibiotics as well as genes associated with intracellular survivability and parasitism (Ripoll *et al.* 2009; Luthra *et al.* 2018). Additionally, *M. abscessus* exhibits two morphological characteristics like smooth phenotype which expresses glycopeptidolipid (GPL) on its cell wall and rough phenotype without GPL expression (Pawlik *et al.* 2013). The virulence potential of rough morphology variant is more than smooth variant whereas rough variant has failed to form biofilm but smooth variant exhibits this feature (Howard *et al.* 2006; Catherinot *et al.* 2009). In our study, we didn’t find any correlation between colony morphology and drug resistance in *M. abscessus* like other studies. This may indicate that the association between drug susceptibility and colony morphology is region specific. Therefore, it is important to explore the genetic composition of two phenotype variants of *M. abscessus*. In this study, we sequenced and analyzed the genomes of Mab^S^ and Mab^R^ strains along with their genome-wide methylation profile. The sequencing result showed that the genome length of Mab^R^ (5075529 bp) is a little longer than that of Mab^S^ (5067231 bp) whereas 5001 and 4963 coding genes are found in Mab^R^ and Mab^S^ respectively. This variation may be due to recombination and/or metabolic adaptations in *M. abscessus* clinical isolates. The structural variation analysis revealed that the genome of Mab^R^ varies from Mab^S^ and reference stain genomes. There are 13 insertions and 8 deletions found in the different location in the genome of Mab^R^ with respect to the reference strain. On the other hand, only one insertion was identified in Mab^S^ genome when compared with the reference strain. Interestingly, the divergence was observed in Mab^R^, 7 insertions and 10 deletions in comparison to Mab^S^. Therefore, *M. abscessus* genome is not conservative which shows a continuation of additional genetic material transfer in this species.

The ratio of core/pan was calculated as 0.47195 which shows that the core genome is the ∼47.195% of the pan-genome. This indicates *M. abscessus* had an open pan-genome which will continue to receive new genes. This might be speculated that horizontal gene transfer had a crucial role in the evolution of *M. abscessus* species acquiring additional genetic material. Isolates from different geographical locations of the world *i.e.*, from China to USA, Malaysia, and Russia clustered together thus indicating the global dissemination of *M. abscessus*. Pan-genome phylogeny (Figure S3) provides a higher resolution to distinguish between the strains compared to the core phylogeny, as the pan-genome separated closely related strains easily, which was somewhat evolutionary close in the core genome. The variation in genome size and protein coding genes in *M. abscessus* clinical strains make important morphological and physiological differences between the strains and leads to more genetic diversity observed in the natural environment.

In this study, we identified a range of SNVs by comparing the two clinical strains against the reference strain ATCC 19977^T^. It was observed that Mab^R^ harbored more SNVs than Mab^S^, indicating the existence of extensive genomic modifications in Mab^R^. Notably, 26 SNVs were detected in the inter-genic regions of Mab^R^ whereas no SNVs were found in these regions of Mab^S^. We have identified nsSNVs within the *MAB_1137c* and *MAB_2106c* common to both strains encoding membrane protein, MmpL family and lipoprotein LppL respectively. The MmpL family proteins and lipoprotein LppL play a major role in mycobacterial virulence and host-pathogen interaction, respectively (Sutcliffe and Harrington 2004; Bernut *et al.* 2016). This may be presumed that the genes carrying the SNVs may cause the alternation of characteristics of this NTM. Further study is needed to explore the role of the genes carrying nsSNVs by site directed mutational analysis which will help to understand the alternation overall phenotype, antibiotic resistance mechanism in *M. abscessus*.

The CRISPR/CRISPR-associated (Cas) elements act as a bacterial defense system which recognizes the foreign invaders (viruses and plasmid) and inactivate them (Wiedenheft *et al.* 2012). There were no CRISPRs elements identified in any clinical *M. abscessus* strain as shown in a previous study (Wee *et al.* 2017). The other species of mycobacteria like *M. tuberculosis* CCDC5079 and *M. africanum* GM041182 have 8 and 7 predicted CRISPRs, respectively (Wee *et al.* 2017). It is possible that lack of CRISPR associated defense system in *M. abscessus* causes the genome insertion which is shown in our study and other related studies (Davidson *et al.* 2014).

One of the findings of our study is the identification of 15210 bp linear natural plasmid (pMabS_GZ002) in Mab^S^ but not in Mab^R^ whereas a circular plasmid was detected in *M. abscessus* ATCC 19977^T^ reference strain (Ripoll *et al.* 2009). The pMabS_GZ002 plasmid encodes putative resolvase/invertase/recombinase, relaxase and *mer* operon that may be involved in conjugation, metabolism process, and resistance to organo-mercury compounds as well as the exchange of genetic material may occur either directly or indirectly with other mycobacteria species. However, there is no study reported about the role of linear plasmid in genetic exchanges in *M. abscessus*, so it is important to elucidate the conjugative ability of this mycobacterial linear plasmid in this species of mycobacteria. Moreover, 4mC methylated bases were detected in the plasmid pMabS_GZ002 in this study which is the first report about the mycobacterial natural plasmid harbored methylated bases. The native plasmid doesn’t contain any putative DNA methyltransferase, so the bases methylated in the plasmid are probably by the DNA methyltransferase encoded in the genome. Previous studies reported the circular plasmid in *M. abescessus* (Ripoll *et al.* 2009; Davidson *et al.* 2014; Leão *et al.* 2013) but to our best knowledge no linear plasmid identified in this NTM species before. Further study is required to determine the existing role of this plasmid in *M. abscessus* isolates which can be used as an epidemiological marker. Despite confirmation through assembly and gel electrophoresis, additional analysis may be required to confirm that the plasmid isolated from Mab^S^ strain has linear conformation.

This study also describes the first report of complete methylome analysis of two *M. abscessus* clinical isolates (smooth and rough phenotype) using SMRT sequencing technology. The genome of Mab^S^ was found to be more highly methylated than Mab^R^, around two-fold higher. The identified methylated modifications may protect the genome from damage and regulate the target gene expression. It was found that 4mC modification was higher than that of 6mA in both *M. abscessus* isolates. Previously, it was reported that 6mA modification type was found to be higher in 12 *Mycobacterium tuberculosis* complexes (MTBC) (Zhu *et al.* 2016). Though these two mycobacterial species have higher G+C contents, their methylation patterns were very different. The difference in methylation pattern might be playing a crucial role in the virulence potential of both strains as a previous report showed that rough phenotype variant is more virulent than smooth variant (Catherinot *et al.* 2009). However, our study didn’t focus on global gene expression as well as virulence property of both clinical strains, so it is important to explore correlation between methylome and virulence property of these clinical strains reported here. Interestingly, it was found that the Mab^S^ has more specific modified bases in the genome than Mab^R^ whereas 1807 modified bases detected that common to both clinical strains (Figure 3). We identified three methylated motifs “VVGGCCB”, “VSGGCCKVNB” and “GTNNBVNB” in Mab^S^ strain whereas only one motif “VVGGCCKS” detected in rough strain. The motifs identified in theses strains were different from other mycobacterial species (Zhu *et al.* 2016), which indicates DNA methylation patterns are species-specific and even strain-specific.

The important findings of our study are the identification of methylation variation in *M. abscessus* genes encoding antibiotic-modifying/targeting enzymes and genes responsible for intracellular survival (Table S2). It was observed that the genes encoding antibiotic-modifying/targeting enzymes in Mab^S^ have higher rates of methylation modification than that in Mab^R^ (Table S2), which may indicate that regulation of their expression is different in both strains. Recently, Laencina *et al.* (2018) reported the importance of ESX-4 locus in *M. abscessus* and elucidated their vital role in intracellular survival and pathogenic potential. it was observed that most of the genes of this locus have methylation sites at different positions and some genes (*MAB_3757* and *MAB_0628*) are highly methylated in Mab^S^ whereas we didn’t get such methylation sites in those particular genes in Mab^R^ (Table S2). The previous study has shown that down regulation of the msp1-msp2-gap operon (GPL locus) in three rough variants compared to in a smooth variant (Pawlik *et al.* 2013). This encouraged us to investigate the variation in methylation in this operon in both clinical isolates. We observed that *msp1* and *msp2* are more methylated in Mab^S^ than in Mab^R^ but we didn’t find any modification in *gap* gene. The finding suggests that difference in methylation pattern in GPL locus might be responsible for differential expression of *msp1*and *msp2* genes in both clinical isolates. However, future study needs to focus on the correlation between methylome and persistent potential as well as virulence of *M. abscessus*.

Additionally, genome analysis of both smooth and rough phenotypes identified 95 and 96 MTases respectively. Despite we reported putative methylation motifs of smooth and rough phenotype in this study, the function of these MTases is not clear along with their recognition sites in both *M. abscessus* clinical strains. The epigenetic regulation of global gene expression in *M. abscessus* is not studied so far whereas it has been reported that DNA methylation facilitates *M. tuberculosis* for survival in hypoxia and a stress condition during infections (Shell *et al.* 2013). Moreover, several research groups reported the role of methylome and MTases in different processes in bacteria such as to survive during antibiotics stress, transportation of ion, host adaptation, environmental and physiological stresses tolerance (Srikhanta *et al.* 2005; Fang *et al.* 2012; Srikhanta *et al.* 2009). As we found the variation in methylome profile in both clinical strains, there is a possibility of MTases activity is also different in both strains which need to be studied. We suggest that that MTases might have an important role in virulence, and different environmental stress conditions to protect *M. abscessus*. However, there is not any study report which indicated the role of methylome in this species. Therefore, further study is required to explore the role of methylome in *M. abscessus*.

In conclusion, we have detected and analyzed the genome-wide methylomes of two *M. abscessus* clinical isolates by using SMRT DNA sequencing technology. It was observed that the methylation profiles of both strains were different. The comparative genomic study has shown that both strains have variation in the genome level. This study also provides a comparison of genome polymorphism between two clinical strains of *M. abscessus*. Many key mutations were identified in the important genes that may play a crucial role in tolerance of antibiotic and environmental stress which need to further study. The results of our study raised several questions regarding the functions and importance of methylation sites at genome levels along with the significant role of individual DNA MTases in *M. abscessus*. Our study will provide a better understanding of the impact of methylation on *M. abscessus* virulence and evolution. Furthermore, *M. abscessus* methylome can be considered as a target for alternative strategy to increase the antibiotic efficacy against this emerging pathogen.
